# Mental Health: Molding a Link to Depression

**DOI:** 10.1289/ehp.115-a536a

**Published:** 2007-11

**Authors:** Carol Potera

The physical consequences of living in a damp, moldy house are well documented and include increased asthma attacks and other respiratory ailments, headaches, fatigue, and sore throats. People who live in moldy environments may also have more depression, finds a study of 5,882 adults living in 2,982 households, published in the October 2007 issue of the *American Journal of Public Health*.

The connection between mold and mental health surprised even the lead author, epidemiologist Edmond Shenassa of Brown University, who was skeptical of the mold–depression link suggested by smaller studies. “We thought that once we statistically accounted for physical factors like crowding and psychological aspects like not having control over one’s living environment, then the association between mold and depression would vanish,” he says. But rather than debunking the notion, Shenassa found an association between mold toxins and depression.

Shenassa and colleagues analyzed data collected by the Large Analysis and Review of European Health Status, a survey of housing, health, and place of residence compiled by the WHO in 2002 and 2003. WHO interviewers visited households in eight European cities and asked residents about depressive symptoms, such as problems sleeping and decreased appetite. They also asked whether a physician had diagnosed depression in the past year. Then they measured the level of dampness and mold in each residence and classified any discernable mold exposure as minimal, moderate, or extensive.

About 40% of the residents lived in visibly damp, moldy households, and overall their risk for depression averaged 34–44% higher than that for residents of mold-free dwellings, with moderate exposure associated with the highest increase in risk. Shenassa says there may be a tipping point where a certain critical amount of mold triggers a response that is not dose-related.

The heightened depression risk also correlated to respondents’ perceptions that a damp, moldy environment cannot be controlled, as well as to documented physical health problems linked to mold exposure. “If you are sick from mold and feel you can’t get rid of it, it may affect your mental health,” says Shenassa, who is undertaking animal studies to investigate whether mold toxins alter behavioral and biochemical brain pathways involved in depression.

Robert Gifford, a psychology professor at the University of Victoria, British Columbia, interprets the results cautiously. Considering only the highest level of mold contamination, when both physical health and perception of control were factored in, the link between mold and depression shrank to “virtually nothing,” he says. However, at minimal and moderate mold exposure, even when controlling for both mediators, there still remained a statistically significant 28–34% higher risk, says Shenassa.

“There is a small relationship between [depression and] mold and dampness, but it is impossible to say that there is a causal relationship,” Gifford says. In addition, more details about income should be explored—wealthier people can afford to clean up extensive mold contamination, whereas low-income people may be forced to live with it. “Income could be an important missing variable,” he notes.

